# Diagnostic and therapeutic challenges of an ambiguous cystic kidney disease in a resource limited setting: a case report

**DOI:** 10.1186/s13104-017-2437-8

**Published:** 2017-03-01

**Authors:** Christian Akem Dimala, Ndemazie Nkafu Bechem, Benjamin Momo Kadia, Vitalis Fambombi Feteh, Simeon Pierre Choukem

**Affiliations:** 1 Faculty of Epidemiology and Population Health, London School of Hygiene and Tropical Medicine, London, UK; 2 Orthopaedics Department, Southend University Hospital, Essex, UK; 3 Health and Human Development (2HD) Research Group, Douala, Cameroon; 4 Penka Michel District Hospital, Penka-Michel, Cameroon; 5 Presbyterian General Hospital Acha-Tugi, Acha, Cameroon; 6 Mboppi Baptist Hospital, Douala, Cameroon; 7 Faculty of Health Sciences, University of Buea, Buea, Cameroon; 8 Douala General Hospital, Douala, Cameroon

**Keywords:** Unilateral renal cystic disease, Multicystic dysplastic kidney, Diagnosis, Cameroon

## Abstract

**Background:**

Unilateral renal cystic disease is a rare condition that shares morphological similarities with multicystic dysplastic kidney, the former often distinguished from the latter on some clinical and histopathological grounds. However serious diagnostic and therapeutic dilemmas set in when there is a considerable overlap in the distinguishing features between these entities.

**Case presentation:**

A 19-year-old African female presented with a chronic severe debilitating right lower quadrant abdominal pain refractory to analgesics. Biochemical investigations and imaging studies revealed a non-functional polycystic right kidney and no identifiable pelvicalyceal system or ureter but with preserved renal function. The marked overlap in clinical presentation between unilateral renal cystic disease and multicystic dysplastic kidney in this patient necessitated further investigation to pose an appropriate diagnosis. A right nephrectomy was performed and histopathological analysis of the resected kidney done, the results of which were more consistent with unilateral renal cystic disease. The post-operative course was favorable.

**Conclusion:**

Unilateral renal cystic disease with an ipsilateral non-functional kidney and an atretic pelvicalyceal system is a very rare condition that needs to be distinguished from multicystic dysplastic kidney in order to guide management and set prognosis. A suspicion of either of these diseases therefore warrants a thorough clinical evaluation and the appropriate combination of biochemical and imaging investigations.

## Background

Unilateral renal cystic disease (URCD) is a rare condition characterized by replacement of the renal parenchyma by multiple cysts without involvement of the contralateral kidney [1]. Few URCD cases have been reported worldwide and none so far in Cameroon. URCD is very similar to multicystic dysplastic kidney (MCDK), a form of renal dysplasia characterized by multiple cysts studded on the dysplastic parenchyma of the non-functional kidney [2]. MCDK is relatively more frequent with a reported incidence of 1 in 3600 live births in its unilateral presentation and 1 in 4300 live births in the bilateral and unilateral presentations combined [3]. In addition to their remarkable similarities in morphology and to some extent their clinical presentations, URCD and MCDK both have a benign natural history and no genetic background [4–8]. This makes the distinction between both renal pathologies challenging especially when there is significant overlap between their distinguishing features. However, differentiating between them is pivotal in deciding on the most appropriate management option. We report the case of an ambiguous polycystic renal pathology primarily diagnosed as URCD but with some features of MCDK. The subsequent management challenges are also discussed.

## Case presentation

**Fig. 1:**
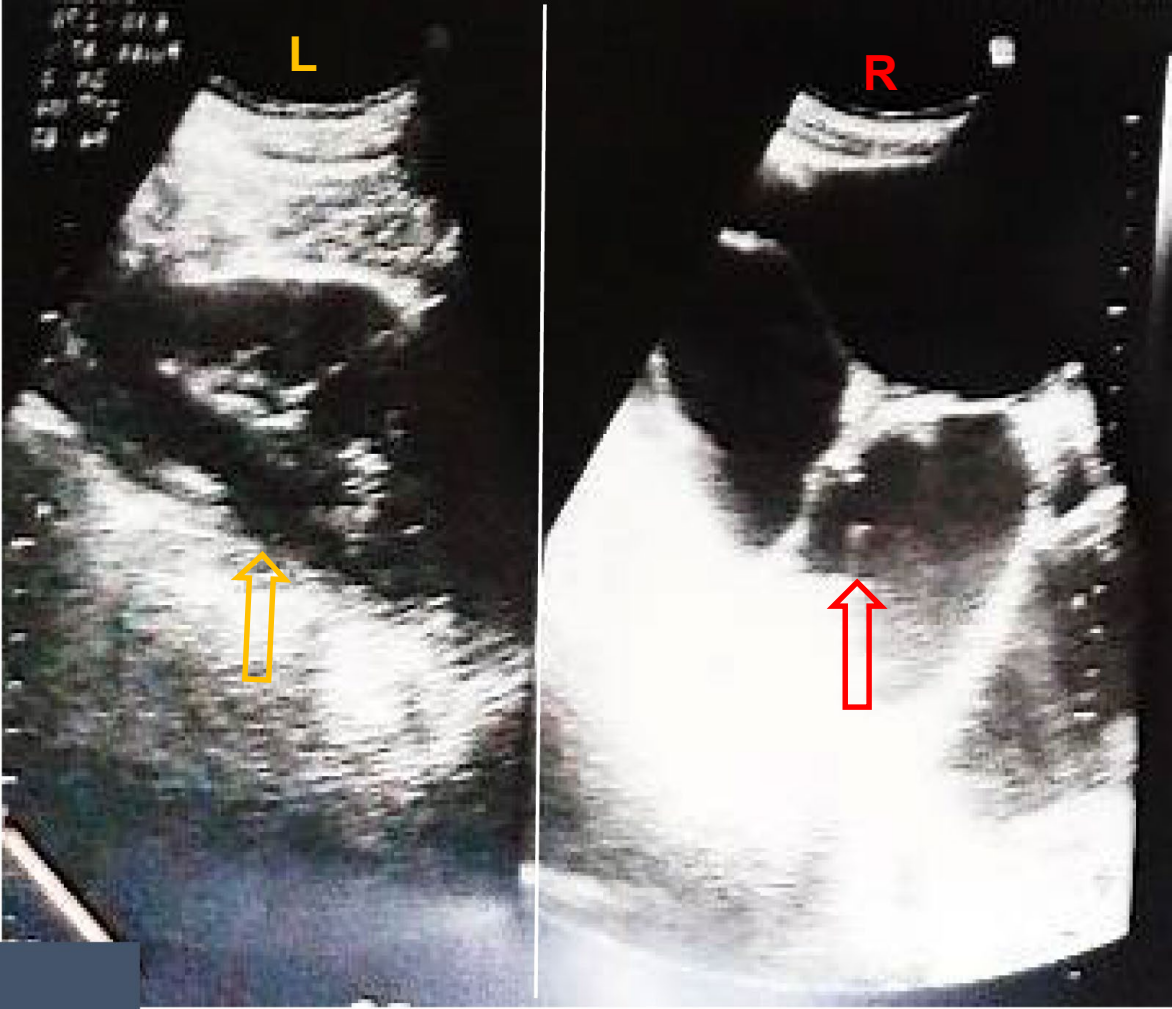
Abdominal ultrasound showing the left (L) and the right (R) kidneys. The left kidney is normal (*yellow arrow*) while the right kidney has multiple fluid filled cysts (*red arrow*)

**Fig. 2:**
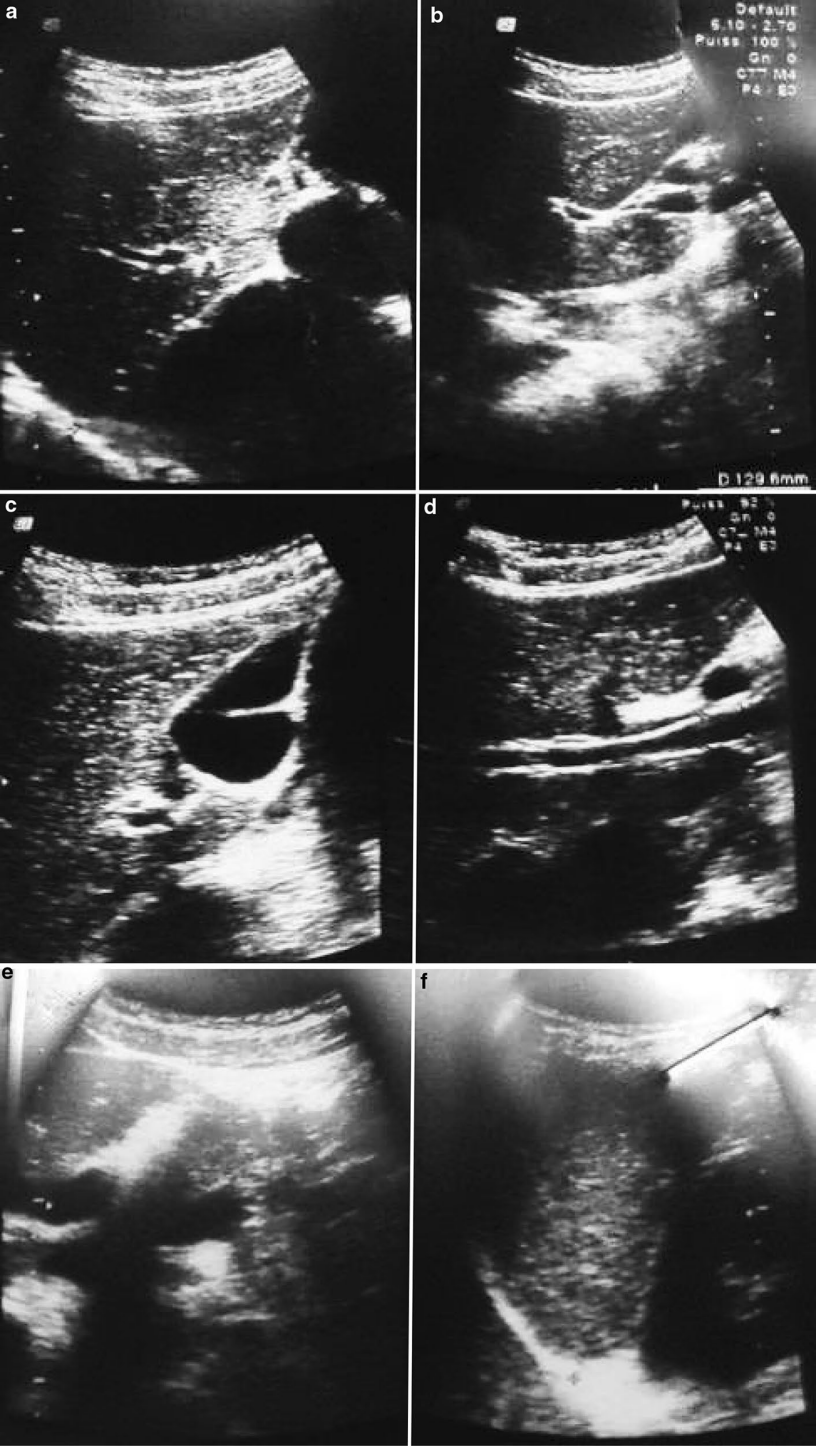
Abdominal ultrasound showing the other abdominal viscera. The right (**a**) and left (**b**) liver lobes, the gall bladder (**c**), pancreatic duct (**d**), the pancreas (**e**) and spleen (**f**) are all normal. No viscus anomaly detected

**Fig. 3:**
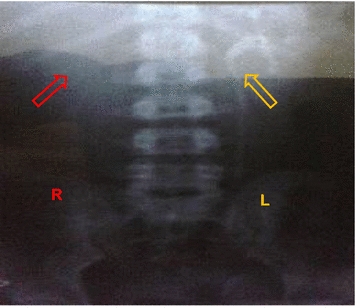
Intravenous urography showing left (L) and right (R) sides of the abdomen. The left renal calyx and ureter (*yellow arrow*) are visible while the right renal calyx and ureter are not visible as expected (*red arrow*)

A 19-year-old African female presented to us with severe and progressively debilitating right iliac fossa pain of 1 month duration. The pain was persistent, progressively increasing in intensity, gnawing in character, aggravated by movement and relieved by lying with thighs flexed. There was no radiation or migration of this pain. She had associated polyuria, increased urinary frequency and nocturia but neither dysuria nor hematuria. There was no urinary incontinence, hesitancy, urgency or urethral discharge. She reported recurrent episodes of fever since the onset of the abdominal pain for which she self-medicated with acetaminophen. There was no vomiting, no change in appetite or bowel habits and no reported change in weight. She had no previous history of surgery, was sexually active, nulliparous and her last normal menstrual period was about 2 weeks prior to consultation. On physical examination her blood pressure and other vital signs were normal. She had an asymmetric abdominal distension, tender at the right lumbar and iliac fossa regions with right costovertebral angle tenderness. A palpable mass was felt in the right iliac fossa, it was immobile, tender with ill-defined borders. There was rebound tenderness at the right iliac fossa. A diagnosis of an appendiceal abscess with an associated urinary tract infection was made and corresponding investigations ordered to exclude other differential diagnoses such as pelvic inflammatory disease. Her white cell count was normal but the lymphocytes were a little raised, and the other parameters were unremarkable (white blood count-8300/mm^3^, granulocytes-44.6%, lymphocytes-47%, monocytes-7%, hemoglobin-11.2 g/dL, hematocrit-39.9%, platelets-327.000/mm^3^). Urine dipstick was normal and urine microscopy was unremarkable. Renal function tests were normal (urea: 5.0 mg/dL; creatinine: 1.1 mg/dL, glomerular filtration rate using the Cockcroft-Gault Equation: 91 mL/min), human immunodeficiency virus, hepatitis C Virus, hepatitis B surface antigen, chlamydia, gonorrhoea and syphilis tests results were all negative. Abdominal ultrasound revealed an enlarged right kidney with multiple large cysts over the entire renal parenchyma (Fig. 1) that fulfilled the criteria suggested by Ravine and colleagues for diagnosis of cystic kidney disease [7]. No cysts or abnormalities were observed in other intra-abdominal organs (Fig. 2). An intravenous urography done showed no contrast material in the right renal collecting ductal or pelvicalyceal systems and ureter, suggestive of a non-functional right kidney. The contrast material was present in the left renal collecting ductal and pelvicalyceal systems and ureter which were otherwise normal (Fig. 3). She was programmed for a surgical exploration that found a huge right kidney with multiple cysts for which a right nephrectomy was done. Postoperatively, the patient was placed on anti-inflammatories, analgesics and antibiotics and the evolution was favorable with normal clinical and renal function parameters. The patient was discharged 5 days after surgery and reviewed 1 week later. The histopathology analysis of the resected kidney revealed cystically dilated tubules with some intervening renal parenchyma and no malignant involvement, all in favor of a renal polycystic disease, however, the non-availability of an electronic database for the storage of patient medical records and data in this resource-limited setting hindered the later retrieval of the histopathological slides. The patient was advised about the need for an initial regular follow-up to ascertain adequate renal function and proper functioning of the remaining kidney.

## Discussion

The overall rarity of reported URCD cases and the diagnostic and therapeutic dilemmas that arise in situations of atypical clinical presentations and similarities to other renal pathologies such as MCDK as observed in this case, make this review particularly relevant. The clinical picture of URCD in this patient was confounded by the additional characteristic atretic pelvicalyceal system observed in MCDK, warranting the use of histopathology to ascertain diagnosis.

URCD is a renal pathology wherein there is a unilateral replacement of the renal parenchyma by several cysts but with no association to other genetic cystic diseases. The pathogenesis of URCD is still not clearly understood, but it has been hypothesized that URCD arises from a developmental anomaly similar to that observed in autosomal-dominant polycystic kidney disease with which it shares remarkable pathological similarity [9]. Despite the few cases of URCD observed in children and neonates, this renal pathology has been predominantly reported in adulthood [4]. MCDK, with a well-documented pathogenesis on the other hand, is a form of renal dysplasia wherein there are multiple cysts studded on the dysplastic renal parenchyma. MCDK results from an abnormal metanephric differentiation often on a background of an obstructive uropathy [2]. This probably explains its frequent early detection, pre-natally or in infancy [2, 4] and its male preponderance [10]. However, the late clinical presentation in this case was not sufficient to rule out MCDK which has also been diagnosed in adulthood in some instances [2, 4]. The absence of a family history of renal disease or deaths due to a renal condition in this case is consistent with both renal pathologies which are non-hereditary [10, 11]. Nevertheless, cases of MCDK inheritance though few have earlier been discussed [4, 8]. With progression to adulthood, MCDK is usually asymptomatic as opposed to URCD which usually presents with flank or abdominal pain, a palpable abdominal mass, hematuria and exclusive unilateral involvement of the kidney. This patient, with no relevant past medical history, presented with severe debilitating abdominal pain, intermittent fever, some urinary symptoms and a right iliac fossa mass. Both disorders can also present with hypertension [2, 4] which was, however, not present in this patient. The asymptomatic presentation of MCDK later in life could be due to the spontaneous partial or complete involution of the kidney [2, 12] due to the resorption of the fluid in the cysts [2, 3]. It is suggested that this cyst involution occurs alongside that of the renal parenchyma making the affected kidney undetectable in half of MCDK cases above 5 years of age [13]. In addition to being mainly asymptomatic, MCDK is almost always unilateral when detected in adulthood since the bilateral presentation, though more common, is generally fatal in infancy [2]. This unilateral presentation is often associated with several extra-renal anomalies in other organs and body systems [2], including contralateral renal abnormalities in more than a quarter of the cases [3]. No intra-abdominal organ or contralateral renal anomaly was detected on abdominal ultrasound in this patient making a diagnosis of MCDK further unlikely.

A closer look at the macroscopic renal morphology shows that URCD consists of multiple cysts of varying sizes diffusely studded on the renal medulla and cortex with a functional calyceal system, in contrast to the cysts in MCDK which are generally fewer and predominantly cortical in distribution [4, 10]. However, the URCD and MCDK overlap in presentation in this case is further accentuated by the complete absence of the contrast material in the right ureter on intravenous urography, suggestive of an obstructed or atretic right pelvicalyceal system or ureter. This distinguishing feature has rather been documented in MCDK, in which there is usually an under-developed or absent pelvicalyceal system, ureters or renal vessels. This developmental anomaly could extend to the lower moiety of the renal tree with consequent ureteropelvic atresia in more extreme situations [2, 4]. In addition to this, an earlier report suggests that MCDK cases with renal pelvis atresia are unlikely to present with contralateral urinary abnormalities [14], implying the contralateral renal sparing which is typical of URCD [15] and which was observed in this patient is less helpful in posing a definitive diagnosis. Nevertheless, the expected compensatory hypertrophy of the contralateral kidney in MCDK [16] was, however, not seen in this patient. The overlap in clinical presentation and macroscopic renal features in this patient brings up diagnostic dilemmas imposing histopathology as a mandatory investigation to differentiate between these two entities. The cystic dilations interspersed by renal parenchyma as found on histopathology of the resected kidney were more suggestive of a URCD rather than of a renal dysplasia. We did not perform a voiding cystourethrography (VCUG) of the contralateral kidney to check for any vesicoureteral reflux to further rule out MCDK since the benefits of this invasive procedure are yet to be proven [2]. Moreover, neither hydroureter nor hydronephrosis (both pointers towards a reflux nephropathy) were observed on abdominal ultrasound.

Possible complications of URCD such as cysts rupture or infection, as was probably the case in this patient, have been reported [17]. The presence of intractable severe debilitating pain on a background of intermittent fever both suggestive of a probable complication, together with the non-functional nature of the kidney therefore mandated nephrectomy. The importance of a correct diagnosis in this situation cannot be over-emphasized since the conservative management often recommended in MCDK [12] does not always apply to polycystic kidney diseases such as URCD, for which surgical nephrectomy becomes a management option in situations of failed conservative management such as cyst decortication and non-responsiveness to analgesics [18]. Mindful of the normal renal function test results on admission, it is worth mentioning that URCD typically does not progress to end stage renal failure. However, patient follow-up remains important to ascertain normal renal function over the years and prompt treatment of any pathologies that could ultimately compromise the optimal functioning of the remaining kidney.

## Conclusions

This case represents a diagnostic dilemma because of the borderline and atypical historical, clinical and laboratory features of URCD and MCDK making the management challenging. That notwithstanding, URCD is displayed here as a rare but important differential diagnosis for subacute abdominal pain at any age and its diagnosis demands a high index of suspicion, thorough clinical assessment together with laboratory and imaging investigations so as to confidently rule out similar renal pathologies.

## Abbreviations

MCDK: multicystic dysplastic kidney
URCD: unilateral renal cystic disease
VCUG: voiding cystourethrography
